# Effects of Early Continuous Venovenous Hemofiltration on E-Selectin, Hemodynamic Stability, and Ventilatory Function in Patients with Septic-Shock-Induced Acute Respiratory Distress Syndrome

**DOI:** 10.1155/2016/7463130

**Published:** 2016-12-01

**Authors:** Jian-biao Meng, Zhi-zhen Lai, Xiu-juan Xu, Chun-lian Ji, Ma-hong Hu, Geng Zhang

**Affiliations:** Department of Intensive Care Unit, Tongde Hospital of Zhejiang Province, 234 Gucui Road, Hangzhou 310012, China

## Abstract

*Objective*. To investigate the effects of 72-hour early-initiated continuous venovenous hemofiltration (ECVVH) treatment in patients with septic-shock-induced acute respiratory distress syndrome (ARDS) (not acute kidney injury, AKI) with regard to serum E-selectin and measurements of lung function and hemodynamic stability.* Methods*. This prospective nonblinded single institutional randomized study involved 51 patients who were randomly assigned to receive or not receive ECVVH, an ECVVH group (*n* = 24) and a non-ECVVH group (*n* = 27). Besides standard therapies, patients in ECVVH group underwent CVVH for 72 h.* Results*. At 0 and 24 h after initiation of treatment, arterial partial pressure of oxygen/fraction of inspired oxygen (PaO_2_/FiO_2_) ratio, extravascular lung water index (EVLWI), and E-selectin level were not significantly different between groups (all *P* > 0.05). Compared to non-ECVVH group, PaO_2_/FiO_2_ is significantly higher and EVLWI and E-selectin level are significantly lower in ECVVH group (all *P* < 0.05) at 48 h and 72 h after initiation of treatment. The lengths of mechanical ventilation and stay in intensive care unit (ICU) were shorter in ECVVH group (all *P* < 0.05), but there was no difference in 28-day mortality between two groups.* Conclusions*. In patients with septic-shock-induced ARDS (not AKI), treatment with ECVVH in addition to standard therapies improves endothelial function, lung function, and hemodynamic stability and reduces the lengths of mechanical ventilation and stay in ICU.

## 1. Introduction

Acute respiratory distress syndrome (ARDS) is characterized by protein-rich pulmonary edema due to the accumulation of extravascular lung water (EVLW), related to increased permeability of alveolar epithelium and endothelial injury in pulmonary vessels [1]. ARDS develops as a complication of several acute disease processes, with sepsis being the most common predisposing condition [2]. ARDS remains the leading complication accounting for the morbidity and mortality of patients with severe sepsis and septic shock. Although the last several decades have witnessed the progress in supportive care strategies for ARDS, the ARDS-associated mortality and morbidity remain high, especially in patients with severe sepsis and septic shock [3].

Both cellular and humoral inflammatory responses are involved in the pathogenesis of ARDS and sepsis. One of the cellular response processes, which involve neutrophils, macrophage/monocytes, lymphocytes, and activated endothelial cells, is the expression of cell adhesion molecules (selectins and integrins) [4, 5].

E-selectin, which belongs to a family of adhesion molecules, is only expressed in activated endothelial cells and involved in leukocyte-endothelial adhesion. E-selectin reflects the endothelial change and probable mechanism in ARDS patients.

Since sepsis and ARDS can coexist and have similar vascular injuries, elevated E-selectin levels are significantly more likely to cause ARDS and also significantly associated with increased 28-day mortality [6]. This indicates that endothelial cells and their components are potential targets for therapeutic intervention in patients with ARDS and sepsis.

An increase in EVLW is the pathophysiological characteristic of hydrostatic pulmonary edema and ARDS [7]. EVLW is also high in many septic shock patients and other critically ill patients [8]. The emergence of transpulmonary thermodilution has opened up the era of EVLW investigation in clinic. According to the physiology, EVLW may rise as a result of increased pulmonary microvascular hydrostatic pressure, or decreased blood colloid osmotic pressure, or as typically in ARDS, aggravated permeability of the alveolocapillary barrier. As reported, at any given pulmonary microvascular hydrostatic pressure in ARDS, a larger increase in pulmonary microvascular permeability is related with the greater outward fluid filtration from microvessels [9]. Therefore, the increase in EVLW indicates the increased impairment of pulmonary permeability.

Continuous renal replacement therapy (CRRT) is widely used in critically ill patients with a beneficial therapeutic effect [10]. Although CRRT has been extensively used as renal support for critically ill patients in intensive care unit (ICU) [10], nonrenal indications for CRRT have been reported recently [11]. Although the therapeutic mechanisms of CRRT for illnesses without acute kidney injury (AKI) or renal failure are not cleared adequately, CRRT for nonrenal indications has been studied in patients with severe sepsis/septic shock [12, 13], ARDS [14], and severe acute pancreatitis [15]. In addition, application of CRRT to children with septic shock or maple syrup urine disease is simple and safe and contributes to the management of various illnesses without renal failure [16]. CRRT can decrease E-selectin production, improve pulmonary permeability, and protect endothelial function in patients with severe acute pancreatitis (SAP) [17]. It also can decrease EVLW in ARDS patients and improve the outcomes of critically ill patients [18].

EVLW reflects pulmonary permeability and the serum level of E-selectin can be used to assess the endothelial function in continuous venovenous hemofiltration (CVVH) treated patients; hence, the purpose of the current study is to assess the change of endothelial function in patients with septic-shock-induced ARDS during CVVH. We further hypothesize that early CVVH favorably influences endothelial function through its excellent clinical and immune homeostasis effect.

## 2. Materials and Methods

### 2.1. Ethics Statement

This pilot study was conducted in accordance with the Helsinki Declaration and the protocol approved by the Ethics Committee of Tongde Hospital of Zhejiang Province (reg. number [2013] 051-048). All participants were recruited by Tongde Hospital of Zhejiang Province and they all (or their guardians) provided written informed consents to participate.

### 2.2. Patients

A total of 119 consecutive patients with infection were admitted to the 20-bed ICU at Tongde Hospital of Zhejiang Province, between November 2014 and March 2016. The exclusion criteria were age < 18 years, pregnancy, absence of fluid resuscitation, unstable hemodynamic condition (change in vasoactive drug dosage or fluid administration within 1 h preceding the protocol), pneumonia, acute coronary syndrome, and uncontrolled tachyarrhythmia (heart rate ≥ 140 beats/min). Patients who died within 48 h after the implementation of the Pulse Indicator Continue Cardiac Output (PiCCO) system or patients with preexisting AKI were also excluded. The diagnosis of septic shock was defined according to the Surviving Sepsis Campaign criteria: International Guidelines for Management of Severe Sepsis and Septic Shock 2012 [19]. Patients who developed septic shock and second onset of ARDS within the preceding 48 h were considered eligible. ARDS was defined according to the criteria recommended by the Berlin definition [20].

**Figure 1:**
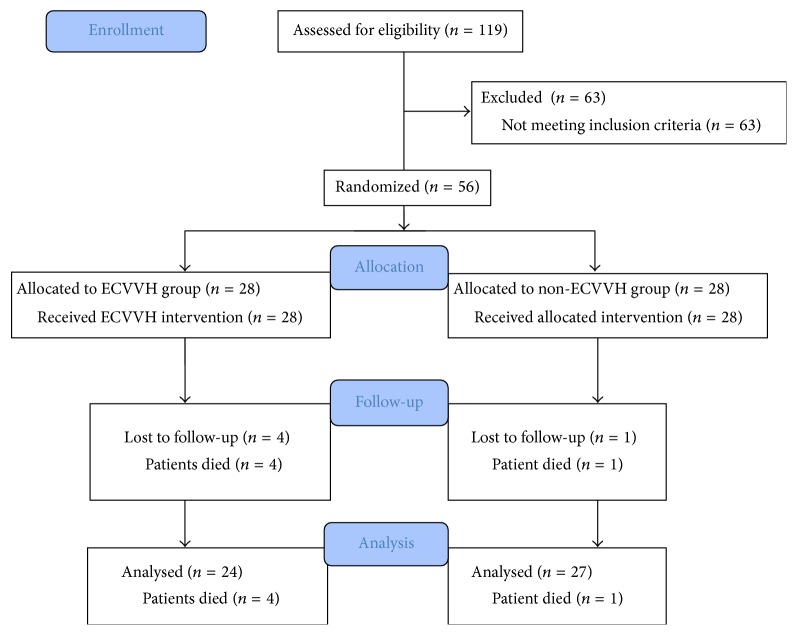
Flow chart of patient enrollment.

Of these 119 patients, we excluded 35 patients without septic shock at admission and 28 patients without septic-shock-induced ARDS. The remaining 56 patients were randomly assigned to receive or not receive early-initiated continuous venovenous hemofiltration (ECVVH) (initiated within 24 h after onset of septic-shock-induced ARDS): an ECVVH group (*n* = 28) and a non-ECVVH group (*n* = 28). The details of the recruitment process are shown in Figure 1.

### 2.3. Treatment Protocol

According to International Guidelines for Management of Severe Sepsis and Septic Shock 2012 [19], all of patients were given conventional treatment method. After a central venous catheter and a radial artery catheter were placed into the patients, we used norepinephrine to maintain a mean arterial pressure (MAP) of at least 65 mmHg despite quantitative fluid resuscitation (the variation of central venous pressure (CVP) from 12 to 15 mmHg). With central venous oxygen saturation (ScvO_2_) at <70% and hematocrit (HCT) at <30%, red blood cells were transfused until HCT was ≥30%. In the case that ScvO_2_ was <70%, dobutamine was applied until ScvO_2_ was ≥70%.

All of the patients were provided with midazolam 0.1 mg/kg/h, sufentanil 0.2 *μ*g/kg/h, and atracurium 0.3 mg/kg/h for sedation, analgesia, and neuromuscular blockade, respectively, if necessary.

They were intubated and received mechanical ventilation, using volume-assist-control mode, with a tidal volume of 6 mL per kilogram of predicted body weight (Table 1). We maintained the airway pressure measured after a 0.5-second pause at the end of inspiration, and plateau pressures were measured with a half-second inspiratory pause. Ratio of the duration of inspiration to the duration of expiration was set at 1 : 2 and ventilator rate setting needed to achieve a pH goal of 7.25 to 7.45, and a lower or higher than normal partial pressure of carbon dioxide (PaCO_2_) can be accepted, left to the discretion of the attending physician. Respiratory-system compliance was calculated as the tidal volume divided by the difference between the peak inspiratory pressure (PIP) and positive end-expiratory pressure (PEEP). The fraction of inspired oxygen (FiO_2_) and PEEP were set to maintain an arterial partial pressure of oxygen (PaO_2_) >60 mmHg. Patients were weaned and extubated according to the standard protocol described in the ARDSNet study [21].

For each patient in the ECVVH group, a double lumen catheter was inserted percutaneously into the right femoral vein to establish vascular access. CVVH was conducted on a PRISMA CRRT machine (Hospital Gambro Co., Melsungen, Germany) equipped with an AN69 membrane (surface area 1.5 m^2^; Gambro Industries, France; changed every 12 h). The blood flow was 180 to 220 mL/min, and the prediluted (before the hemofilter) replacement solution was infused at a rate of 35 mL/kg·h. Low-molecular-weight heparin was used for anticoagulation of the circuit in patients without coagulopathy, and ultrafiltrate was removed at a rate of 200 mL/hr. Replacement solutions consist of Na^+^ 147 mmol/L, Cl^−^ 115 mmol/L, HCO_3_^−^ 36 mmol/L, Ca^2+^ 2.4 mmol/L, Mg^2+^ 0.7 mmol/L, and Glu 10.33 mmol/L. K^+^ was adjusted accordingly (Chengdu Qingshan Likang Pharmaceutical Co., Ltd. China). CVVH was initiated within 24 h after onset of septic-shock-induced ARDS.

All patients were monitored using a transpulmonary thermodilution device (PiCCO, Pulsion Medical System, Munich, Germany). They were inserted with both a left femoral artery catheter and a right central venous catheter in place. The correct placement of the catheter for insertion was further confirmed by chest radiography.

A 5-French thermistor-tipped catheter (PV2014L16, Pulsion Medical System, Germany) was inserted into the femoral artery and a central venous catheter (CS-277202-E, ARROW, US) was placed into a central jugular or subclavian vein; both were connected to the PiCCO system. Thermodilution parameters and pulse contour parameter were recorded by the PiCCO monitor, based on triplicate injections of 15 mL of 0.9% cold isotonic saline (<8°C) via the central venous catheter, and were expressed as the average of three measurements. The corresponding ventilator function and perfusion parameters were observed and kept constant during the 6 h period preceding the measurements. The patients were kept in the horizontal position.

### 2.4. Measurement of Arterial Blood Gas

Blood gases from the arterial catheters were sampled anaerobically in 3 mL heparinized syringes (PL67BP; BD Diagnostics, Plymouth, UK) and analysed on a blood gas bedside machine (ABL800: Radiometer, Copenhagen, Denmark).

### 2.5. Plasma E-Selectin Concentration

Blood samples were collected from a preexisting arterial cannula at 0, 24, 48, and 72 h after the start of treatment and immediately centrifuged. The serum was stored at −70°C until used. The E-selectin levels were measured in sera via enzyme-linked immunosorbent assay (ELISA) on an Evidence Investigator E-Selectin High-Sensitivity Array system (Jingmei Bio Engineering Co. Ltd., Shenzhen, China) according to the manufacturer's protocol.

### 2.6. Data Collection

Demographic characteristics including age, sex, EVLW index (EVLWI), and PaO_2_/FiO_2_ were reviewed. Sequential organ failure assessment (SOFA), multiple organ dysfunction score (MODS), and acute physiology and chronic health evaluation (APACHE II) scores were recorded. E-selectin, ventilator function, hemodynamic parameters, fluid balance, and serum creatinine at 0, 24, 48, and 72 h after the start of treatment were recorded.

The experimental procedure and laboratory results of the study were not concealed to treating physicians.

### 2.7. Statistical Analysis

PASS software (version 11; NCSS, LLC.) was used for calculate sample size. Sample size was determined by Two-Sample *t*-Test Power Analysis using preliminary data obtained in our laboratory with the following assumptions: *α* of 0.05 (two-tailed), power of 80%, the difference in the mean of E-selectin at 72 h between patients in ECVVH and non-ECVVH group of −14.7 ng/mL, and a standard deviation of 2.3 ng/mL. Therefore, we calculated that a sample size of 23 would have an 80% power of detecting a difference at a level of significance of 0.05.

Data were expressed as mean ± standard deviation of the mean (SDM) for quantitative variables and as count and percentages for qualitative values. Distributions of the discrete variables were compared between the 2 treatment groups with either the Chi-square test or Fisher exact tests. Two-sample *t*-test was used to compare between the two groups and paired *t*-test to compare continuous variables before and after treatment. SPSS software (version 16; SPSS Inc., Chicago, IL) was used for statistical analysis; all tests were 2-tailed and *P* < 0.05 was considered to be statistically significant.

## 3. Results

### 3.1. Patients

Of these 56 patients, 4 patient died in the ECVVH group and one patient died in the non-ECVVH group within 48 h after recruitment and we excluded them for incomplete data. Hence, 51 patients with complete data were enrolled in this study (Figure 1). Demographic and clinical data from the 24 patients in ECVVH group and 27 patients in non-ECVVH group are summarized in Table 1. The demographic data are not significantly different between ECVVH group and non-ECVVH group: 62.8 ± 16.4 versus 58.6 ± 17.8 years old (age; *P* = 0.39) and 16 versus 17 males (*P* = 0.78). The clinical data before treatment were not significantly different between the ECVVH group and non-ECVVH group: 13.2 ± 2.8 mL/kg versus 12.8 ± 3.1 mL/kg (EVLWI; *P* = 0.63) and 165.1 ± 61.2 mmHg versus 158.8 ± 54.7 mmHg (PaO_2_/FiO_2_; *P* = 0.70). The assessment scores at admission were not significantly different between the ECVVH group and non-ECVVH group: 19.7 ± 4.9 versus 21.1 ± 5.7 (APACHE II scores; *P* = 0.36); 8.6 ± 0.7 versus 8.3 ± 0.6 (MODS scores; *P* = 0.11); 7.1 ± 0.8 versus 6.8 ± 0.6 (SOFA scores; *P* = 0.13). The average initial serum creatinine was 0.79 ± 0.23 mg/dL versus 0.82 ± 0.27 mg/dL (*P* = 0.67) and the lactate levels were 2.8 ± 0.8 mmol/L versus 2.5 ± 0.7 mmol/L (*P* = 0.16) in the ECVVH group and non-ECVVH group. The sites of infection which contributed to ARDS in all patients included intra-abdominal, skin, bloodstream, and urinary tract and none of them showed significant difference. There was no difference in categories of ARDS between two groups.

### 3.2. Ventilatory Function and PaO_2_/FiO_2_ and Their Changes after Treatment

The ventilator function parameters and PaO_2_/FiO_2_ are listed in Table 2. There are no significant differences in PaO_2_/FiO_2_, dynamic compliance (Cdyn), plateau pressure (Pplat), and PEEP before treatment between groups (*P* = 0.70, 0.38, 0.65, and 0.53, resp.). After treatment, although PaO_2_/FiO_2_ tended to increase in both groups, it increased significantly at both 48 and 72 h in ECVVH group versus non-ECVVH group (*P* = 0.01, <0.001, resp.). During the course of treatment, Cdyn increased gradually while Pplat decreased in two groups, but Cdyn increased more significantly at 48 and 72 h in ECVVH group versus non-ECVVH group (*P* = 0.02, 0.00, resp.) and Pplat decreased more significantly at 48 and 72 h in ECVVH group versus non-ECVVH group (*P* = 0.03, <0.001, resp.). In addition, PEEP also decreased more significantly at 72 h in ECVVH group versus non-ECVVH group (*P* = 0.04). There are no significant differences in pH and PaCO_2_ before and after treatment between groups (all *P* > 0.05).

### 3.3. Hemodynamics Parameters, Serum Creatinine, and Fluid Balance and Their Changes after Treatment

The hemodynamic parameters are listed in Table 3. There were no significant differences in intrathoracic blood volume index (ITBVI), cardiac index (CI), mean arterial pressure (MAP), and systemic vascular resistance index (SVRI) before treatment (0 h) between groups (all *P* > 0.05). Despite no significant difference in EVLWI, after treatment it decreased significantly at 48 and 72 h in ECVVH group versus non-ECVVH group (*P* = 0.01, 0.04, resp.). ITBVI, MAP, and SVRI in two groups tended to increase after treatment, but none of them differed significantly at any time point between groups (all *P* > 0.05). CI in two groups tended to decrease after treatment but did not differ significantly at any time point between groups (all *P* > 0.05). Compared to non-ECVVH group, ECVVH showed more benefits to fluid removal in ARDS patients at 24, 48, and 72 h after treatment (*P* < 0.001). Furthermore, the doses of norepinephrine in ECVVH group were improved more significantly at 48 and 72 h of treatment (both *P* < 0.001).

### 3.4. Changes in E-Selectin Level before and after Treatment

As shown in Table 4, the E-selectin levels in patients with septic-shock-induced ARDS are remarkably high but are not significantly different between two groups (*P* = 0.33). During the course of treatment, E-selectin levels tended to decrease in both groups and significantly at 48 and 72 h in ECVVH group versus non-ECVVH group (*P* = 0.04, <0.001, resp.).

**Figure 2:**
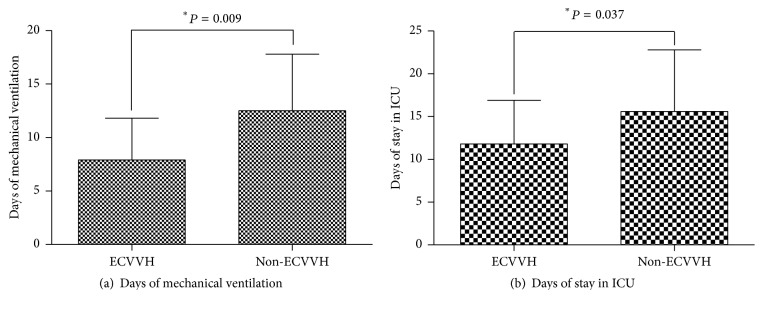
Comparison of days of mechanical ventilation (a) and stay in ICU (b) between ECVVH and non-ECVVH groups.

We demonstrated that patients with ECVVH required less days of mechanical ventilation (7.9 ± 3.9 days versus 12.5 ± 5.3 days, resp., *P* = 0.01) and stay in ICU compared with those without CVVH (11.8 ± 5.1 versus 15.6 ± 7.2, resp., *P* = 0.04) (Figure 2). However, the overall mortality at day 28 did not show significant difference between two groups (25.0% versus 29.6%, resp., *P* = 0.71).

## 4. Discussion

ARDS is closely associated with sepsis, especially septic shock, and commonly coexists with septic shock. We find that ECVVH not only could decrease EVLWI or E-selectin level and improve dynamic compliance (Cdyn) and oxygenation, but has no adverse effect on hemodynamics in patients with septic-shock-induced ARDS.

ARDS is characterized by noncardiogenic pulmonary edema due to the accumulation of EVLW. EVLW reflects pulmonary edema and plays prognostic roles for patients with septic-shock-induced ARDS and correlates with the severity of ARDS. EVLWI is normally between 3 and 7 mL/kg and a level above 10 mL/kg is associated with clinical pulmonary edema [22]. Therefore, reducing EVLW or balancing intravascular volume expansion against the negative effects of pulmonary edema induction might be critical for patients with septic-shock-induced ARDS [23].

It is widely recognized that defective fluid clearance is associated with compromised alveolar-capillary barrier function in ARDS, and edema fluid must be cleared for ARDS patients to survive [24]. CRRT, one of extracorporeal treatments, has been used for critically ill patients with or without AKI or acute kidney failure and ARDS is one of nonrenal indications for CRRT [11]. Studies showed that CRRT brought benefits to ARDS patients (non-AKI), including improvement of oxygenation and survival [14, 25]. We used CVVH, one of CRRT, in the current study and we found significant improvement of EVLWI in both groups after initiation of treatment. CVVH patients showed lower EVLWI than non-CVVH patients at 48 and 72 h of treatment. Our results confirm the beneficial effects of CVVH on the clinical outcomes in patients with septic-shock-induced ARDS.

Although CVVH has many benefits to critically ill patients, especially when applied early [26], the effects of early CVVH remain unclear in patients with septic-shock-induced ARDS. We compared early initiation of conventional treatments (within 24 hours after onset of ARDS) with CVVH versus conventional treatments without CVVH and found early CVVH significantly improved clinical outcomes. Many studies indicate early CRRT has many benefits to critically ill patients. A septic shock piglet model shows that conventional CVVH in the early stage of septic shock could effectively improve hemodynamics and oxygen metabolism and eliminate blood inflammatory mediators [27]. An experimental animal model of endotoxin-induced septic lung injury confirms the beneficial effects of CVVH on gas exchange and lung mechanics [27]. Early CVVH can attenuate the entire inflammatory response and improve arterial oxygenation and lung mechanics, including peak inspiratory pressure and lung compliance [28]. In addition to these animal studies, there are some studies presenting the beneficial effects of early CVVH on critically ill patients. Early CRRT was found as an independent factor associated with a lower mortality rate, the severity of disease, and causative organisms in the patients with septic acute kidney injury [29]. Early CRRT helps to reduce EVLWI, improve PaO_2_/FiO_2_ and Cdyn, and shorten duration on ventilation in ARDS patients [25]. In this study, we find significant improvements in EVLWI, PaO_2_/FiO_2_, Cdyn, Pplat, and PEEP, which are consistent with these studies.

Several possible mechanisms have been proposed to explain the improved arterial oxygenation and lung mechanics in patients with septic-shock-induced ARDS after treatment with CVVH. First, CVVH could contribute to fluid removal by achieving a negative fluid balance [30]. Increased EVLW worsened lung edema and predicted mortality in severe sepsis and septic shock [31, 32] and aggravated ALI/ARDS [7]. A negative fluid balance was found to be associated with decreased EVLW [33]. Negative fluid balance contributed not only to PaO_2_/FiO_2_, as well as lower Pplat and PEEP [34], but also to decreases in mechanical ventilation times and mortality [35]. ECVVH reached the goal of negative fluid balance in the early stage of septic shock, and EVLW could be decreased through the fluid removal during CVVH. Recent clinical and animal studies showed that CVVH was associated with improved oxygenation and organ function, facilitated fluid removal, and decreased EVLW in ALI/ARDS patients [25, 28]. Many cytokines and biomarkers, including endothelial and epithelial alveolar biomarkers, involved in the pathogenesis of ARDS and sepsis are released and it is believed that CVVH attenuates the entire inflammatory response and reduces the levels of biomarkers via removal of cytokines [14, 36].

E-selectin belongs to a family of membrane-bound glycoproteins only present in activated endothelial cells and is involved in leukocyte-endothelial adhesion especially in response to inflammation [37]. E-selectin level elevation is associated with sepsis and higher mortality [38]. Since sepsis and ALI/ARDS have similar vascular injuries, Okajima et al. measured plasma E-selectin levels in 50 patients who presented with evidence of systemic inflammatory response syndrome and found that higher E-selectin level is significantly associated with the development of ALI/ARDS and higher 28-day mortality [6].

We found endothelial dysfunction and higher levels of E-selectin in patients with septic-shock-induced ARDS. The E-selectin levels tend to decrease in all patients, but at 72 h after start of CVVH, these changes are significantly different between ECVVH patients and non-ECVVH patients. It might indicate that early CVVH can attenuate endothelial dysfunction in patients with septic shock-induced ARDS.

Since E-selectin (molecular weight about 85–105 kDa) cannot be directly removed by convection and diffusion during CVVH, it is not reasonable to assure that the decrease in E-selectin levels is due to removal by CVVH. However, our study suggests CVVH may promote a decrease in E-selectin levels. This effect may be attributed to several explanations. Since the AN69 membrane used for CVVH in our study was changed every 12 h, absorptive removal of this molecule cannot be excluded. In addition to this mechanism, calcium acts as a universal second messenger in various cells including endothelial cells and regulates numerous gene expressions (including E-selectin) and protein synthesis pathways. Decreased concentration of endothelial intracellular Ca^2+^ after CVVH indicated that the removal of inflammatory cytokines by CVVH may diminish the expression of E-selectin [17]. Nevertheless, further studies are needed to clarify the underlying mechanisms.

We also examined hemodynamic parameters in these patients. Interestingly, MAP was not decreased but remained more stable during treatment in two groups, as evidenced by the more reduced dose of norepinephrine in ECVVH group, suggesting no negative or aggravated signs in hypotension or hemodynamic parameters during CVVH. On the other hand, the norepinephrine dose improvement indicates the beneficial effects of CVVH on tissue hypoperfusion. In addition, the stable ITBVI, CI, and SVRI and the decrease in norepinephrine dose demonstrate improved fluid responsiveness in the ECVVH group. CVVH stabilized hemodynamics in critically ill patients [39], and the effect could be associated with the suppression of inflammation and the removal of cytokines [14].

However, this study has two limitations. First of all, we chose change in E-selectin as the primary endpoint of this study. In our study, the number of patients with septic-shock-induced ARDS was small and the study period was relatively brief; therefore, we should take the risk of positive results into consideration in a study with numerous secondary variables. Second, our study did not show a statistically significant difference in 28-day mortality between both groups and its difference was 4.6%, suggesting that the effect size was much lower than that assumed in the sample size calculation. Post hoc sample size calculations show that ≥1471 patients per group would be required to show a statistically significant difference in 28-day mortality between two groups. It could not be achieved in a single center and a multicenter study would be required in the future.

## 5. Conclusions

In patients with septic shock-induced ARDS and normal kidney function, early treatment with CVVH in addition to standard therapy ameliorates endothelial dysfunction, improves lung function and hemodynamic stability, and reduces the length of mechanical ventilation and length of stay in the ICU.

## Figures and Tables

**Table 1:** Demographic and clinical characteristics between ECVVH and non-ECVVH groups.

Characteristic	ECVVH (*n* = 24)	non-ECVVH (*n* = 27)	*P* value
Age, y	62.8 ± 16.4	58.6 ± 17.8	0.39
Male (*n*, (%))	16 (67)	17 (63)	0.78
EVLWI (mL/kg)	13.2 ± 2.8	12.8 ± 3.1	0.63
PaO_2_/FiO_2_ (mmHg)	165.1 ± 61.2	158.8 ± 54.7	0.70
APACHE II	19.7 ± 4.9	21.1 ± 5.7	0.36
MODS	8.6 ± 0.7	8.3 ± 0.6	0.11
SOFA	7.1 ± 0.8	6.8 ± 0.6	0.13
Serum creatinine (mg/dL)	0.79 ± 0.23	0.82 ± 0.27	0.67
Lactate (mmol/L)	2.8 ± 0.8	2.5 ± 0.7	0.16
Site of infection (*n*, (%))			
Intra-abdominal	13 (54.2%)	13 (48.1%)	0.67
Skin	3 (12.5%)	4 (14.8%)	0.81
Bloodstream	5 (20.8%)	6 (22.2%)	0.90
Urinary tract	3 (12.5%)	4 (14.8%)	0.81
Categories of ARDS			
Mild ARDS	0 (0%)	0 (0%)	—
Moderate ARDS	19 (79.2%)	23 (85.2%)	0.57
Severe ARDS	5 (20.8%)	4 (14.8%)	0.57

APACHE II = acute physiology and chronic health evaluation II, ARDS = acute respiratory distress syndrome, ECVVH = early continuous venovenous hemofiltration, EVLWI = extravascular lung water index, FiO_2_ = fraction of inspired oxygen, MODS = multiple organ dysfunction score, PaO_2_ = arterial partial pressure of oxygen, and SOFA = sequential organ failure assessment.

**Table 2:** Changes in ventilatory functions between two groups at 0, 24, 48, and 72 h after initiation of treatment.

Parameter	ECVVH group (*n* = 24)	non-ECVVH group (*n* = 27)	*P* value
PaO_2_/FiO_2_ (mmHg)			
0 h	165.1 ± 61.2	158.8 ± 54.7	0.70
24 h	194.7 ± 52.8	174.5 ± 68.3	0.25
48 h	247.1 ± 87.3	191.4 ± 56.7	0.01
72 h	281.3 ± 38.6	234.5 ± 54.2	<0.001
pH			
0 h	7.36 ± 0.07	7.37 ± 0.07	0.61
24 h	7.37 ± 0.07	7.37 ± 0.07	1.0
48 h	7.36 ± 0.07	7.36 ± 0.08	1.0
72 h	7.36 ± 0.07	7.36 ± 0.07	1.0
PaCO_2_ (mmHg)			
0 h	43 ± 12	43 ± 12	1.0
24 h	45 ± 13	46 ± 13	0.79
48 h	46 ± 13	48 ± 14	0.60
72 h	46 ± 12	48 ± 14	0.59
Cdyn (mL/cmH_2_O)			
0 h	23.2 ± 3.3	22.3 ± 3.9	0.38
24 h	26.6 ± 5.6	24.5 ± 4.7	0.15
48 h	33.8 ± 7.5	28.2 ± 8.3	0.02
72 h	35.1 ± 5.7	29.7 ± 6.8	<0.001
Pplat (cmH_2_O)			
0 h	28.2 ± 1.9	27.9 ± 2.7	0.65
24 h	25.3 ± 1.7	25.8 ± 2.3	0.39
48 h	23.4 ± 3.2	25.2 ± 2.6	0.03
72 h	19.7 ± 2.6	22.4 ± 1.9	<0.001
PEEP (cmH_2_O)			
0 h	9.0 ± 3.1	8.5 ± 2.8	0.53
24 h	8.8 ± 2.8	8.5 ± 2.7	0.69
48 h	7.3 ± 2.3	8.3 ± 2.8	0.16
72 h	6.7 ± 2.2	8.0 ± 2.4	0.04

Cdyn = dynamic compliance, ECVVH = early continuous venovenous hemofiltration, FiO_2_ = fraction of inspired oxygen, PaCO_2_ = partial pressure of carbon dioxide, PaO_2_ = arterial partial pressure of oxygen, PEEP = positive end-expiratory pressure, and Pplat = plateau pressure.

**Table 3:** Hemodynamics, serum creatinine, and fluid balance between two groups at 0, 24, 48, and 72 h after initiation of treatment.

Hemodynamic variable	ECVVH (*n* = 24)	non-ECVVH (*n* = 27)	*P* value
Norepinephrine dose (*μ*g/kg/min)			
0 h	0.41 ± 0.12	0.39 ± 0.10	0.52
24 h	0.35 ± 0.11	0.36 ± 0.09	0.72
48 h	0.21 ± 0.07	0.33 ± 0.08	<0.001
72 h	0.06 ± 0.03	0.15 ± 0.05	<0.001
Serum creatinine (mg/dL)			
0 h	0.79 ± 0.23	0.82 ± 0.27	0.67
24 h	0.70 ± 0.21	0.86 ± 0.28	0.03
48 h	0.55 ± 0.18	1.11 ± 0.33	<0.001
72 h	0.54 ± 0.18	1.26 ± 0.41	<0.001
Fluid balance (mL)			
0 h	1189.9 ± 146.5	1129.4 ± 139.9	0.14
24 h	304.8 ± 49.7	426.2 ± 50.6	<0.001
48 h	−509.5 ± 60.8	−300.3 ± 39.7	<0.001
72 h	−407.4 ± 55.8	−196.2 ± 17.8	<0.001
ITBVI (mL/m^2^)			
0 h	623.3 ± 117.9	642.6 ± 87.0	0.52
24 h	981.7 ± 98.8	960.4 ± 112.4	0.48
48 h	971.7 ± 94.5	1023.0 ± 104.1	0.07
72 h	998.2 ± 125.6	951.8 ± 146.5	0.23
CI (L/min/m^2^)			
0 h	5.7 ± 0.5	5.5 ± 0.9	0.34
24 h	5.1 ± 0.8	4.8 ± 1.4	0.36
48 h	4.3 ± 0.7	4.5 ± 1.1	0.45
72 h	4.4 ± 0.4	4.5 ± 0.7	0.54
MAP (mmHg)			
0 h	75.2 ± 9.3	72.6 ± 10.4	0.35
24 h	83.6 ± 7.9	81.1 ± 9.8	0.48
48 h	89 ± 6.8	86.1 ± 8.1	0.18
72 h	88.1 ± 8.2	85.4 ± 7.4	0.22
SVRI (dyns·s/(cm^5^·m^2^))			
0 h	1117 ± 383	1065 ± 482	0.67
24 h	1317 ± 309	1289 ± 360	0.77
48 h	1662 ± 274	1584 ± 267	0.31
72 h	1683 ± 224	1641 ± 282	0.56
EVLWI (mL/kg)			
0 h	13.2 ± 2.8	12.8 ± 3.1	0.63
24 h	12.5 ± 3.4	12.7 ± 3.6	0.84
48 h	9.1 ± 2.5	11.0 ± 2.7	0.01
72 h	7.6 ± 2.8	9.4 ± 3.2	0.04

CI = cardiac index, ECVVH = early continuous venovenous hemofiltration, EVLWI = extravascular lung water index, ITBVI = intrathoracic blood volume index, MAP = mean arterial pressure, and SVRI = systemic vascular resistance index.

**Table 4:** Circulating level of E-selectin between two groups at 0, 24, 48, and 72 h after initiation of treatment.

E-selectin (ng/mL)	ECVVH (*n* = 24)	non-ECVVH (*n* = 27)	*P* value
0 h	91.1 ± 28.4	84.5 ± 19.2	0.33
24 h	92.7 ± 27.1	93.2 ± 23.6	0.94
48 h	50.7 ± 29.9	67.5 ± 25.8	0.04
72 h	41.7 ± 17.3	60.4 ± 19.7	<0.001

ECVVH = early continuous venovenous hemofiltration.

## References

[1] Ware L. B., Matthay M. A. (2000). The acute respiratory distress syndrome. *The New England Journal of Medicine*.

[2] Hudson L. D., Milberg J. A., Anardi D., Maunder R. J. (1995). Clinical risks for development of the acute respiratory distress syndrome. *American Journal of Respiratory and Critical Care Medicine*.

[3] Rubenfeld G. D., Caldwell E., Peabody E. (2005). Incidence and outcomes of acute lung injury. *The New England Journal of Medicine*.

[4] Bernard G. R., Artigas A., Brigham K. L. (1994). The American-European Consensus Conference on ARDS: definitions, mechanisms, relevant outcomes, and clinical trial coordination. *American Journal of Respiratory and Critical Care Medicine*.

[5] Aird W. C. (2007). Phenotypic heterogeneity of the endothelium: I. Structure, function, and mechanisms. *Circulation Research*.

[6] Okajima K., Harada N., Sakurai G. (2006). Rapid assay for plasma soluble E-selectin predicts the development of acute respiratory distress syndrome in patients with systemic inflammatory response syndrome. *Translational Research*.

[7] Kushimoto S., Taira Y., Kitazawa Y. (2012). The clinical usefulness of extravascular lung water and pulmonary vascular permeability index to diagnose and characterize pulmonary edema: a prospective multicenter study on the quantitative differential diagnostic definition for acute lung injury/acute respiratory distress syndrome. *Critical Care*.

[8] Martin G. S., Eaton S., Mealer M., Moss M. (2005). Extravascular lung water in patients with severe sepsis: a prospective cohort study. *Critical Care*.

[9] Jozwiak M., Teboul J.-L., Monnet X. (2015). Extravascular lung water in critical care: recent advances and clinical applications. *Annals of Intensive Care*.

[10] Ronco C., Bellomo R., Ricci Z. (2001). Continuous renal replacement therapy in critically ill patients. *Nephrology Dialysis Transplantation*.

[11] Oda S., Sadahiro T., Hirayama Y. (2010). Non-renal indications for continuous renal replacement therapy: current status in Japan. *Contributions to Nephrology*.

[12] Peng Y. Z., Yuan Z. Q., Li H. B. (2005). Removal of inflammatory cytokines and endotoxin by veno-venous continuous renal replacement therapy for burned patients with sepsis. *Burns*.

[13] Wu C., Wang X. Y., Yu W. K. (2016). Short-term consequences of continuous renal replacement therapy on body composition and metabolic status in sepsis. *Asia Pacific Journal of Clinical Nutrition*.

[14] Yang W.-M., Hong J., Zeng Q.-Y. (2016). Improvement of oxygenation in severe acute respiratory distress syndrome with high-volume continuous veno-venous hemofiltration. *Global Pediatric Health*.

[15] Oda S., Hirasawa H., Shiga H. (2005). Management of intra-abdominal hypertension in patients with severe acute pancreatitis with continuous hemodiafiltration using a polymethyl methacrylate membrane hemofilter. *Therapeutic Apheresis and Dialysis*.

[16] Kornecki A., Tauman R., Lubetzky R., Sivan Y. (2002). Continuous renal replacement therapy for non-renal indications: experience in children. *Israel Medical Association Journal*.

[17] Chen Z.-H., Liu Z.-H., Yu C., Ji D.-X., Li L.-S. (2007). Endothelial dysfunction in patients with severe acute pancreatitis: improved by continuous blood purification therapy. *International Journal of Artificial Organs*.

[18] Dunham C. M. (2001). Clinical impact of continuous renal replacement therapy on multiple organ failure. *World Journal of Surgery*.

[19] Dellinger R. P., Levy M. M., Rhodes A. (2013). Surviving sepsis campaign: international guidelines for management of severe sepsis and septic shock: 2012. *Critical Care Medicine*.

[20] The ARDS Definition Task Force (2012). Acute respiratory distress syndrome: The Berlin definition. *The Journal of the American Medical Association*.

[21] The Acute Respiratory Distress Syndrome Network (2000). Ventilation with lower tidal volumes as compared with traditional tidal volumes for acute lung injury and the acute respiratory distress syndrome. *The New England Journal of Medicine*.

[22] Michard F. (2007). Bedside assessment of extravascular lung water by dilution methods: temptations and pitfalls. *Critical Care Medicine*.

[23] Mitchell J. P., Schuller D., Calandrino F. S., Schuster D. P. (1992). Improved outcome based on fluid management in critically ill patients requiring pulmonary artery catheterization. *American Review of Respiratory Disease*.

[24] Sznajder J. I. (2001). Alveolar edema must be cleared for the acute respiratory distress syndrome patient to survive. *American Journal of Respiratory and Critical Care Medicine*.

[25] Han F., Sun R. H., Ni Y. (2015). Early initiation of continuous renal replacement therapy improves clinical outcomes in patients with acute respiratory distress syndrome. *The American Journal of the Medical Sciences*.

[26] Honore P. M., Jamez J., Wauthier M. (2000). Prospective evaluation of short-term, high-volume isovolemic hemofiltration on the hemodynamic course and outcome in patients with intractable circulatory failure resulting from septic shock. *Critical Care Medicine*.

[27] Chen W.-M., Lu G.-P., Lu Z.-J., Zhang L.-E. (2013). Effects of high volume hemofiltration on hemodynamics and oxygen metabolism at early stage of septic shock in piglet models. *Zhonghua Er Ke Za Zhi*.

[28] Ullrich R., Roeder G., Lorber C. (2001). Continuous venovenous hemofiltration improves arterial oxygenation in endotoxin-induced lung injury in pigs. *Anesthesiology*.

[29] Oh H. J., Shin D. H., Lee M. J. (2012). Early initiation of continuous renal replacement therapy improves patient survival in severe progressive septic acute kidney injury. *Journal of Critical Care*.

[30] van Bommel E. F. H. (1997). Should continuous renal replacement therapy be used for ‘non-renal’ indications in critically ill patients with shock?. *Resuscitation*.

[31] Martin G. S., Eaton S., Mealer M., Moss M. (2005). Extravascular lung water in patients with severe sepsis: a prospective cohort study. *Critical Care*.

[32] Meng J. B., Hu M. H., Lai Z. Z. (2016). Levosimendan versus dobutamine in myocardial injury patients with septic shock: a randomized controlled trial. *Medical Science Monitor*.

[33] Cordemans C., De Laet I., Van Regenmortel N. (2012). Fluid management in critically ill patients: the role of extravascular lung water, abdominal hypertension, capillary leak, and fluid balance. *Annals of Intensive Care*.

[34] Wiedemann H. P., Wheeler A. P., Bernard G. R. (2006). Comparison of two fluid-management strategies in acute lung injury. *The New England Journal of Medicine*.

[35] Murphy C. V., Schramm G. E., Doherty J. A. (2009). The importance of fluid management in acute lung injury secondary to septic shock. *Chest*.

[36] Journois D., Israel-Biet D., Pouard P. (1996). High-volume, zero-balanced hemofiltration to reduce delayed inflammatory response to cardiopulmonary bypass in children. *Anesthesiology*.

[37] McEver R. P. (2002). Selectins: lectins that initiate cell adhesion under flow. *Current Opinion in Cell Biology*.

[38] Boldt J., Wollbruck M., Kuhn D., Linke L. C., Hempelmann G. (1995). Do plasma levels of circulating soluble adhesion molecules differ between surviving and nonsurviving critically ill patients?. *Chest*.

[39] Riera J. A. S. I., Alted E., Lozano M. J., Pérez J. L., Ambrós A., Caballero R. (1997). Influence of continuous hemofiltration on the hemodynamics of trauma patients. *Surgery*.

